# Machine-learning detection and variability of mesospheric frontal waves observed by VIIRS day/night band

**DOI:** 10.1186/s40623-025-02308-4

**Published:** 2025-11-13

**Authors:** Yuta Hozumi, Jia Yue, Seraj Al Mahmud Mostafa, Chenxi Wang, Jianwu Wang, Sanjay Purushotham, Steven D. Miller

**Affiliations:** 1 Department of Physics, Utah State University, Logan, UT US; 2 Catholic University of America, Washington, DC US; 3 NASA Goddard Space Flight Center, Greenbelt, MD US; 4 Department of Information Systems, University of Maryland, Baltimore County, MD US; 5 Cooperative Institute for Research in the Atmosphere, Colorado State University, Fort Collins, CO US

**Keywords:** Airglow imaging, Machine-learning based wave event detection, Frontal wave

## Abstract

Frontal waves, characterized by sharp boundaries of airglow jump accompanied by following undulations, were detected using machine learning techniques, and their variability was examined. Frontal waves are thought to be manifestations of ducted waves called mesospheric bores or “wall” waves (large-amplitude gravity waves). The YOLOv3 machine learning model, short for “You Only Look Once version 3,” was trained to detect frontal wave events in Day/Night Band (DNB) data from the Visible/Infrared Imaging Radiometer Suite (VIIRS) onboard the Suomi National Polar-orbiting Partnership (Suomi NPP) satellite. The YOLOv3 detector was trained with DNB images, including manually labeled objects of 756 unique frontal waves. The model achieved 83.19% of average precision (AP) for frontal wave event detection during the testing phase. Utilizing the trained model, 1,150 frontal wave events were identified out of all available 515,187 moonless images from Suomi NPP VIIRS/DNB from January 2012 to June 2023. Over the past eleven years, the monthly occurrence of frontal wave events has gradually decreased from approximately 15 in 2012 to around 5 in 2022. Frontal waves exhibit a high occurrence peak at equatorial latitudes and weaker occurrence peaks at winter mid-latitudes. In these regions, the migrating diurnal and semidiurnal tides exhibit large temperature amplitudes, which could create a favorable environment for ducted waves or mesospheric bores, such as a temperature inversion layer. Frontal waves detected in this study show higher occurrences in regions where conditions favor the formation of ducted waves or mesospheric bores.

**Graphical Abstract:**

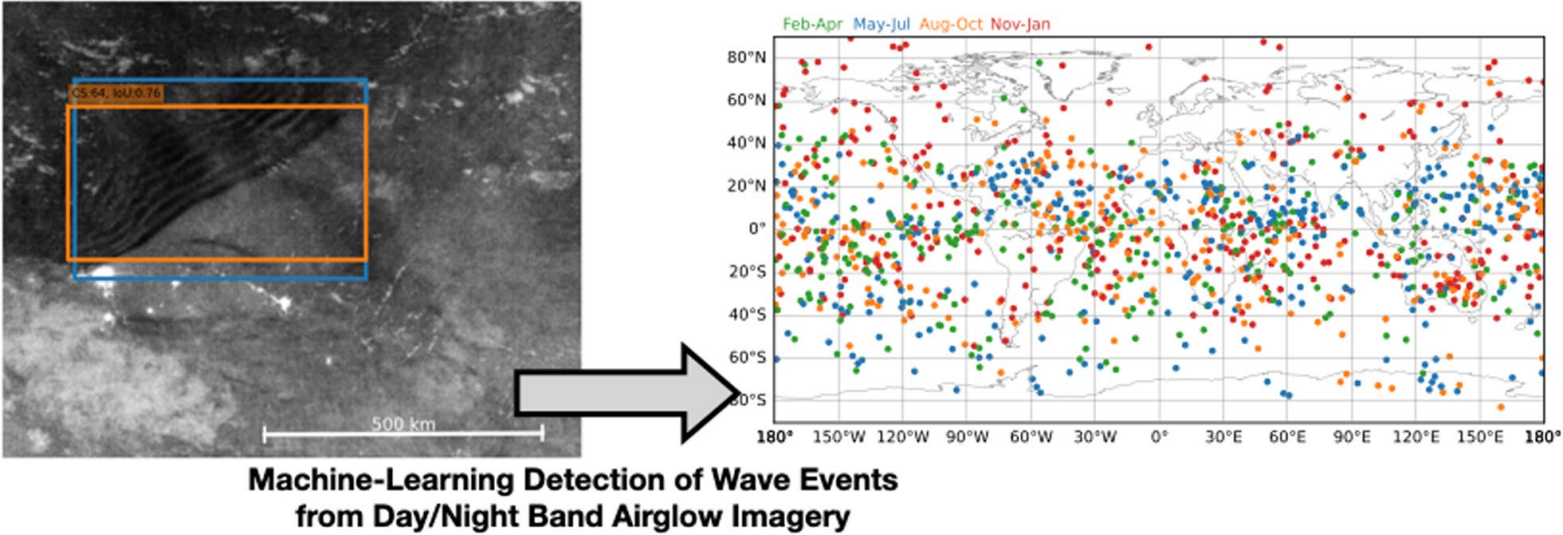

## Introduction

Gravity waves play an essential role in transporting energy and momentum both vertically and horizontally in the Earth’s atmosphere and contribute to maintaining global circulation and thermal structures (e.g., Holton 1982). Space-borne airglow imagers have provided a great opportunity to study gravity waves on a global scale, especially those with short horizontal wavelengths. The Day/Night Band (DNB) on the Visible/Infrared Imaging Radiometer Suite (VIIRS) onboard the Suomi National Polar-orbiting Partnership (Suomi NPP) satellite has the capability to capture mesospheric airglow images. Various airglow signatures related to gravity waves are captured in DNB images, including those generated by orography, convection, intensifying fronts, and volcanic events (Miller et al. 2012, 2013, 2015; Su et al. 2018; Yue et al. 2019). Suomi NPP DNB/VIIRS data have been publicly available since 2012, providing more than eleven years of data. These data enable us to conduct a long-term study on such mesospheric wave activities. However, hundreds of thousands of airglow images have been collected by DNB since 2012, making it impractical to manually identify wave events in such a large dataset. Additionally, due to its broadband sensitivity (505–890 nm, Miller et al. 2013), the data are severely contaminated by emissions from the lower atmosphere, such as city lights, orographic features like snowy mountains, and reflections from cloud tops. This contamination makes it difficult to conduct an automated systematic wave event survey, such as using the variance of airglow perturbations. A novel technique for detecting wave events is essential to maximize the use of the vast DNB dataset and conduct long-term studies.

Recently, machine-learning (ML) techniques have been applied to image analysis in geophysical studies, including gravity waves in airglow (Lai et al. 2019; González et al. 2022), aurora classification (Clausen and Nickisch 2018; Nanjo et al. 2022), and cloud detection (Wang et al. 2020). ML techniques can generate flexible and effective object detectors by learning image patterns from a given dataset. In this study, a wave event detection method was developed using the YOLOv3 (You Only Look Once version 3) model (Redmon and Farhadi 2018), which is a machine-learning-based object detection model. YOLOv3 is widely used in various fields, including agriculture (Lawal 2021) and the medical field (Jiang et al. 2020). This ML-based approach enables us to conduct an efficient and systematic event survey of the extensive DNB dataset. By demonstrating the feasibility of ML for identifying gravity wave events, we aim to establish a foundation for the broader application of these techniques in future analyses. In general, gravity waves exhibit various appearances in airglow, from quasi-monochromatic waves to more chaotic patterns. This study focuses on frontal waves, characterized by a sharp jump in airglow brightness accompanied by following waves. Because frontal waves have one of the most distinct wave signatures among various wave types, they serve well to demonstrate the capability of machine learning in detecting wave events. We developed a frontal wave detector using YOLOv3 and applied the detector to 11 years of DNB data.

Frontal waves have been captured by DNB and they are thought to be mesospheric bores (Miller et al. 2015; Su et al. 2018). Mesospheric bores are propagating discontinuities in a duct (Dewan and Picard 1998, 2001). For ground-based airglow imagers, a bore is observed as a sharp propagating front dividing the nightglow into dim and bright parts (Taylor et al. 1995; Smith et al. 2003, 2005, 2017; Nielsen et al. 2006; Yue et al. 2010). The propagating front is often accompanied by waves or turbulence. Another space-borne airglow sensor, the Visible and near Infrared Spectral Imager (VISI) of the Ionosphere, Mesosphere, Upper Atmosphere, and Plasmasphere (IMAP) mission, has also observed frontal waves, which are thought to be mesospheric bores (Hozumi et al. 2018, 2019). A ducting region, a stable waveguide layer surrounded by less stable or neutrally stable air above and below, such as a temperature inversion layer (a thermal duct) and wind shear (a Doppler duct), is necessary for mesospheric bores. Mesospheric bores and the simultaneous occurrence of ducts have been reported with multi-instrument observations, including optical imaging, lidar, radar, and satellite sounding, and their characteristics have been investigated (She et al. 2004; Smith et al. 2003; Bageston et al. 2011; Fechine et al. 2009; Li et al. 2013, 2019; Hecht et al. 2023). Because ducted waves, such as mesospheric bores, can transport energy over long horizontal distances, understanding their spatial distribution and frequency is essential for comprehending energy transport in the upper mesosphere.

The so-called “wall” wave events in the upper mesosphere can also present a frontal wave appearance (Swenson and Espy 1995; Swenson et al. 1998; Li et al. 2007; Smith 2013). “Wall” wave events are considered to be caused by large amplitude gravity waves that manifest as a steep increase or decrease in airglow along their phase line (Swenson et al. 1998). The sharp boundary caused by large amplitude gravity waves is often accompanied by smaller wave structures. “Wall” waves have a very similar appearance to mesospheric bores; however, this type of wave is not ducted and is a different wave phenomenon from bores. Identifying mesospheric bores and “wall” waves requires additional observations, such as multi-band airglow imaging, lidar, or radar measurements. For single-band imaging like DNB, distinguishing between the two types of waves is challenging. In this study, we treat them together as frontal wave events.

Although frontal waves have been studied extensively using ground-based measurements, these observations are typically confined to specific sites, limiting global context. Satellite data offer the potential for a more comprehensive view, but systematic studies of frontal waves remain limited. Only one prior study by the same authors (Hozumi et al. 2019) used the 3 years of IMAP/VISI to investigate mesospheric bores. However, no long-term climatological investigations of frontal waves have yet been conducted. By applying an ML-based frontal wave detector to 11 years of DNB imagery, we aim to address this gap, providing new insights into where and how often such waves occur on a global scale.

This paper is organized as follows. Section 2 describes the data and preprocessing of DNB images. In section 3, model training is presented. Section 4 presents performance test results. The global distribution and long-time climatology of frontal waves detected by ML are presented and discussed in Sect. 5. Section 6 summarizes the study.

### Data and preprocessing

The Suomi NPP VIIRS DNB was used in this study. Suomi NPP is a sun-synchronous, near-polar satellite (mean altitude $\sim$833 km; inclination 98.79$^\circ$), providing nightside observations with local-time coverage centered near 01:30 LT. Data preprocessing is necessary to convert data into a YOLOv3-compatible format and reduce the data size to conduct training in a realistic time. Data processing was started from level-1B data of DNB. A 6-minute granule of observation data was stored in a raw level-1B data file corresponding to $\sim$3,000 km $\times$ 2,300 km. A data file contains 4,064 $\times$ 3,232 pixel data of 4-byte float type in a unit of Watts/cm$^2$/steradian.

**Fig. 1:**
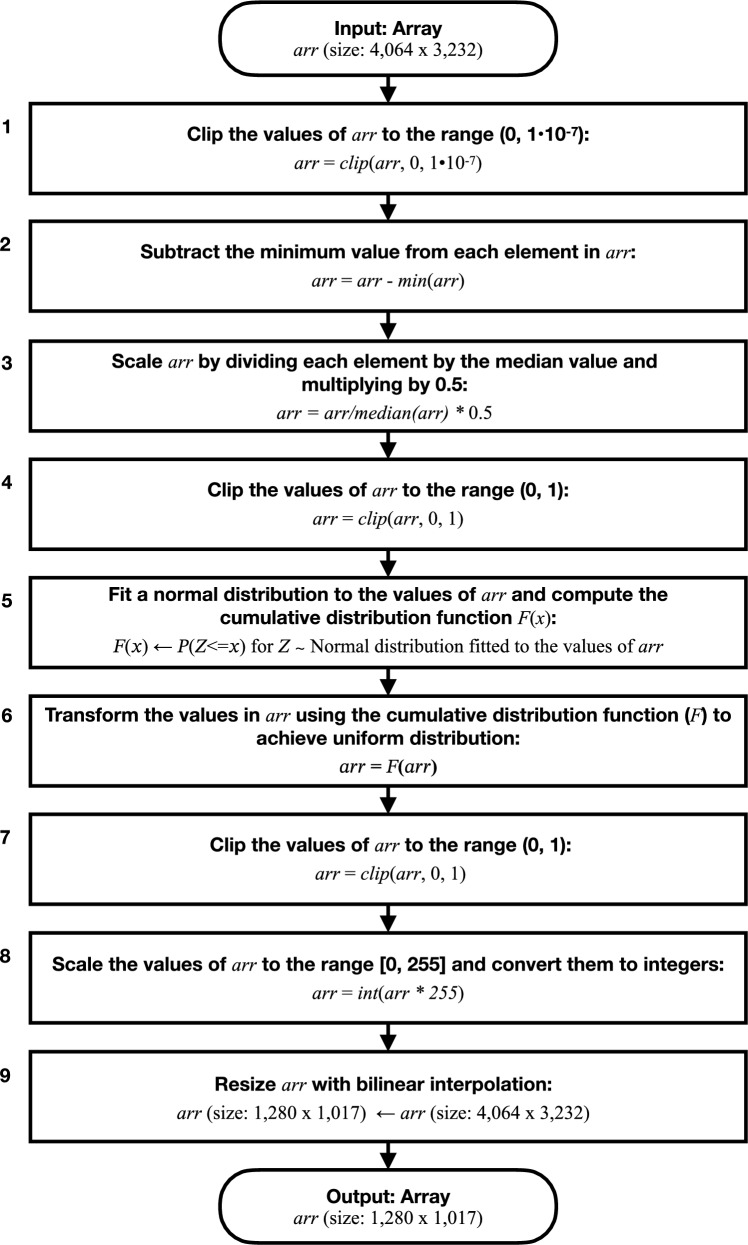
A flowchart of the preprocessing algorithm

Our conversion to an 8-bit (0–255) image follows the flowchart in Fig. 1. First, we clip negative values to 0 and extreme outliers above 1$\times$10$^{-7}$. Such outliers typically result from sensor artifacts rather than meaningful signal; clipping them helps prevent a handful of extreme values from skewing the subsequent median-based scaling. We then subtract the minimum pixel value so that the smallest pixel is 0 and scale by the median to set the median at 0.5. After this step, we clip the data again to [0,1]. This mitigates the impact of large outliers and stabilizes the fitting of the normal distribution (mean and standard deviation) in the subsequent step. Although DNB imagery is not always perfectly normal, applying the fitted cumulative distribution function (CDF) to the data (Step 6) functions similarly to a histogram equalization, enhancing subtle airglow signals by mapping intensities toward a uniform distribution in [0,1]. We clip again if floating-point imprecision puts any values outside [0,1], ensuring a clean range for the final 8-bit conversion.

Finally, we convert the data to 8-bit integers and bilinearly resize it from 4,064 $\times$ 3,232 to 1,280 $\times$ 1,017, resulting in a pixel size of approximately 2.5 km. Overall, these clipping and CDF-transformation steps optimize the dynamic range for airglow detection and reduce the data size for machine learning. Because DNB imagery exhibits a wide range of intensity distributions due to diverse atmospheric and surface conditions, we tested several preprocessing methods. We found that this combination of clipping and CDF-transformation was most effective in enhancing subtle airglow features and preserving relevant details for YOLOv3 training. Note that the per-granule minimum, median, and CDF mapping can be influenced by scene content (e.g., background city lights, cloud-top reflections, thin clouds) as well as instrumental factors. By applying minimum subtraction and median scaling on a per-granule basis, and then mapping values through a fitted CDF, the pipeline largely suppresses additive background offsets and emphasizes the morphological patterns of airglow, though residual sensitivity to scene-composition changes and long-term instrumental state changes may persist.

Only data from moon-free nights (two nights after the last quarter through two nights after the first quarter lunar phase for each lunar cycle) were used in the investigation, owing to the inability to detect airglow features under moonlight conditions (atmospheric and surface scatter overwhelm the signal compared to the relatively weak airglow emissions).

**Fig. 2:**
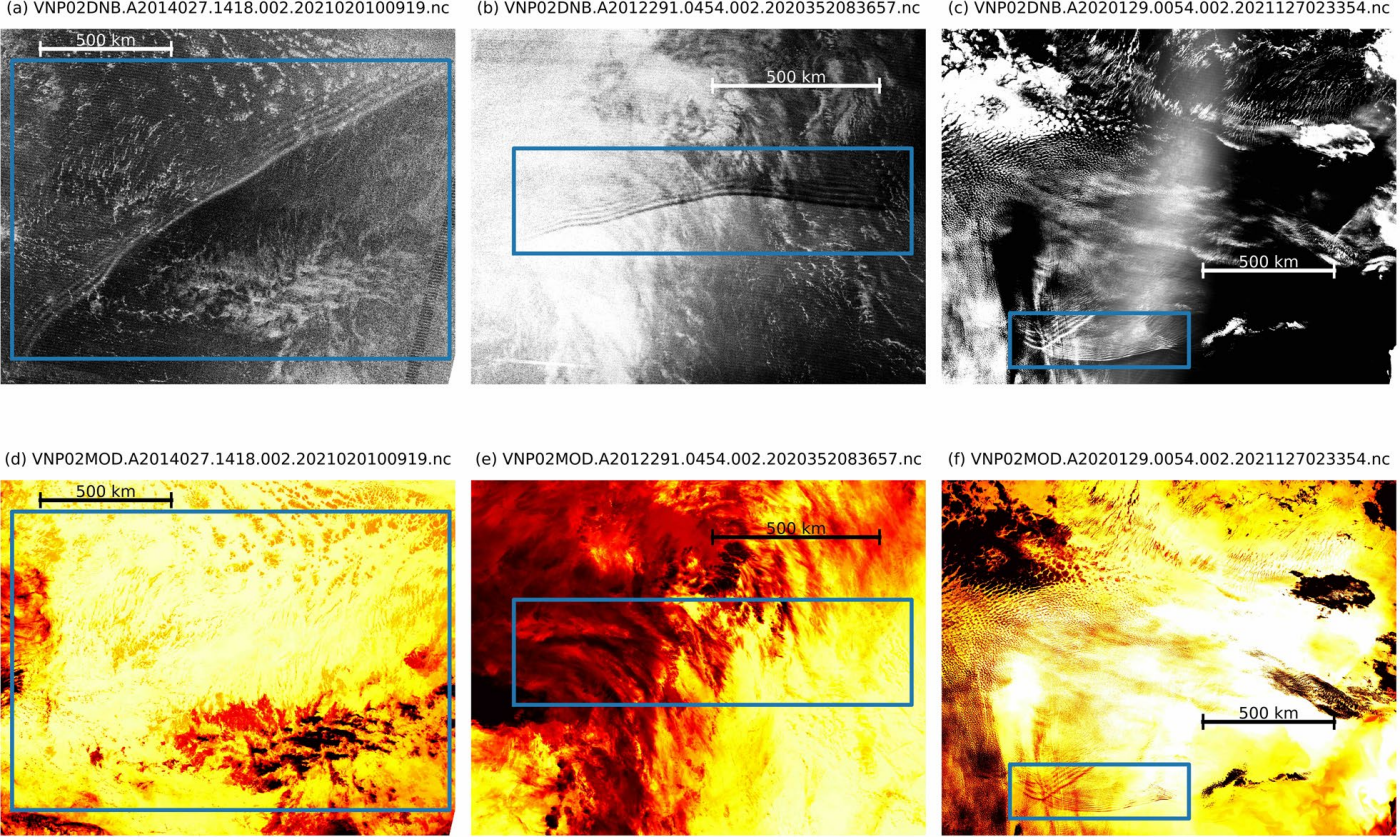
Examples of frontal waves as captured in DNB images: (**a**) a bright jump frontal wave, (**b**) a dark jump frontal wave, and (**c**) a tropospheric frontal wave. These features are highlighted by blue rectangles. (**d**), (**e**) and (**f**), M15 band infrared images of the same areas at the same time as images (**a**), (**b**), and (**c**), respectively, with blue rectangles marking the corresponding location in each image. The name of the original data file is presented on top of each image

A total of 916 DNB images containing 988 frontal wave objects were selected from DNB data from Jan 2013 to July 2023. Some images contain multiple objects, with up to four in a single image. These frontal wave objects were selected based on the following features: (1) a sharp boundary that causes a jump in airglow brightness, and (2) accompanying undulations on only one side of the boundary, with wavefronts parallel to the boundary. Each object was labeled with an axis-aligned rectangular bounding box sized to tightly enclose the full spatial extent of the boundary and the accompanying undulations. We considered both a bright jump accompanied by undulations (on the bright side) and a dark jump accompanied by undulations (on the dark side) as a single frontal wave class. The selection process included both visual inspection by human eyes and detection by a YOLOv3 model we trained in a preliminary study with smaller set of frontal wave images. We found there are some especially sharp frontal wave features in the imagery, which are thought to be tropospheric phenomena manifesting in meteorological clouds. Figure 2 shows examples of a bright jump frontal wave, a dark jump frontal wave, and a tropospheric frontal wave, alongside their corresponding infrared images from the VIIRS M15 band measurement. While the DNB is sensitive to both mesospheric airglow and tropospheric clouds, the M15 infrared measurement is primarily sensitive to tropospheric clouds only. Therefore, features visible in both DNB and M15 images indicate tropospheric cloud features, whereas features only visible in DNB images are attributed to mesospheric airglow. In Fig. 2, each DNB image showcases a sharp brightness jump followed by undulation. However, no corresponding features are observed in the M15 infrared images for Figs. 2(d) and 2(e). In contrast, Fig. 2(f) displays a feature closely correlated with its DNB counterpart (Fig. 2(c)), indicating that the frontal wave feature in Fig. 2(c) is a tropospheric feature. Generally, tropospheric frontal waves are sharper, brighter, and have finer and smaller spatial character than mesospheric frontal waves. We carefully removed this type of tropospheric object from the frontal wave dataset.

The 916 images were randomly divided into 696 for training and 220 for testing. The 696 and 220 image sets contain 756 and 232 frontal waves in total, respectively. To augment the image data set for training, each image was rotated at an angle of 90$^\circ$, 180$^\circ$, and 270$^\circ$, resulting in 2,784 images for training and 880 images for testing. To preserve the image’s structure and orientation in predictable ways, we restricted the rotation angles to these three specific values.

In a preliminary study, we found that a frontal wave ML detector is sometimes tricked into producing false positives by other features like clouds, aurora, or surface features (rivers, ice on the ocean, etc.). To reduce these false positives, 800 negative background images containing aurora, cloud features, etc., but not containing frontal waves, were added to the training data set. In total, the training data set consists of 3,584 images, 2,784 frontal wave images (encompassing 3,024 objects from the original 696 images with 756 objects), and 800 background images. These image sets are provided in https://doi.org/10.5281/zenodo.14812061.

### Model training

YOLO, short for "You Only Look Once," is a clever way of detecting multiple objects in a picture using a neural network. Instead of analyzing the image multiple times, YOLO breaks it into a grid and predicts what objects are in each grid cell. This method is faster than other detectors, making it great for various applications. YOLOv3 strikes a good balance between speed and accuracy, performing well on various benchmark datasets and providing robust detection performance compared to other models (Padmanabula et al. 2020). Unlike two-stage detectors like Faster R-CNN (Region-Based Convolutional Neural Network), YOLOv3 is a single-stage detector, predicting bounding boxes and class probabilities directly from full images in one evaluation, which is computationally efficient. YOLOv3 is simpler, uses fewer resources, and runs faster compared to SSD (Single Shot MultiBox Detector), which are resource-intensive and take much longer to train. Due to its speed, simplicity, and efficiency, YOLOv3 is the right fit for our research objectives.

In YOLOv3, the authors introduced a powerful network called Darknet-53 (Redmon and Farhadi 2018). This network has 53 layers that act as a base for detecting objects. It is faster and more accurate than its predecessor (YOLOv2). The first 53 layers are trained on a task called image classification using the ImageNet dataset. Then, for object detection, 53 more layers are added, totaling 106 layers and creating the final YOLOv3 model. Key elements in the YOLOv3 architecture include residual blocks, skip connections, and upsampling layers. Darknet-53 is efficient, performing billions of operations per second and making better use of the GPU for faster evaluation.

The YOLOv3 process involves two stages: pre-training and detection. In the pre-training stage, the Darknet-53 network learns to recognize objects from the ImageNet dataset. It uses different residual blocks and strided convolutions to capture features and prevent the loss of important details. The network gradually increases the number of filters, and each residual block includes a bottleneck structure. In the detection stage, layers related to image classification are removed, leaving the backbone for object detection. YOLOv3 aims to detect objects at different scales, so detection layers are added after the last three residual groups. This results in three different scales for object detection: small, medium, and large objects. These scales correspond to feature vectors of 52$\times$52, 26$\times$26, and 13$\times$13, respectively. Objects are detected at specific layers (82, 94, and 106) in the network.

YOLOv3 employs a multi-scale approach, enabling it to detect objects of varying sizes within a single pass through its deep neural network. This architecture is well-suited for analyzing the DNB imagery, where frontal waves may exhibit diverse sizes. The utilization of YOLOv3 facilitates the detection of wave events within a huge data set of DNB images.

The model outputs a rectangle bounding box of an object with a confidence score. The confidence score represents the probability that a detected bounding box contains an object of interest. The input resolution of the model is increased to 1,028 $\times$ 1,028 from its original one of 608 x 608 for better performance. We trained the model with the aforementioned 3,584 images for 4,000 epochs, which means that the training dataset was passed through with the model network 4,000 times to update the model’s parameters. The trained weight of the network of YOLOv3 is provided in https://doi.org/10.5281/zenodo.14812061.

### Model performance

#### Test result with labeled images

The performance of the trained model is tested with the 880 images containing 928 frontal wave objects that were not used in training. The threshold of the confidence score is set to 0.2. An object with a confidence score exceeding this threshold is judged as frontal wave detected and vice versa. The evaluation explored the model’s performance across various IoU (Intersection over Union) thresholds, where IoU is defined as the overlap between the detected and manually labeled rectangles. A case in which the IoU exceeds the threshold is judged as TP (true positive), while a case in which the IoU is below the threshold is considered to be that the model fails to identify a correct bore feature and is judged as FN (false negative). Higher IoU means that there is a large overlap between a detected rectangular and a manually labeled rectangular. A higher IoU threshold demands more severe requirements for the model and then leads to degradation of the evaluation parameters, such as fewer TPs and more FNs. The test results are presented in Table 1 FP and AP denote false positive and average precision, respectively. Precision, recall, and the F1 score are expressed as follows.

$$ \textrm{Precision} = \frac{\textrm{TP}}{\textrm{TP} + \textrm{FP}} $$

$$ \textrm{Recall} = \frac{\textrm{TP}}{\textrm{TP} + \textrm{FN}} $$

$$ \mathrm {F_1} = \frac{2}{\frac{1}{\textrm{Precision}} + \frac{1}{\textrm{Recall}}} $$

The average precision is expressed as follows;

$$ \textrm{AP} = \sum _{n}\bigl (R_{n} - R_{n-1}\bigr ) \, P_{n} $$

Where $P_n$ and $R_n$ are the precision and recall at the n-th threshold of confidence level. As the IoU threshold decreases, metrics such as precision, recall, F1 Score, and AP improve. For instance, at an IoU threshold of 0.6, there were 307 TPs with an AP of 30.74%. Reducing the IoU threshold to 0.2 increased the TPs to 575 and the AP to 83.19%. Higher AP at lower IoU thresholds indicates that the model is capable of detecting many true objects but struggles with accurately predicting the size of frontal waves. This difficulty arises due to the fact that the edges of frontal waves are often unclear, and the appearance features of frontal waves gradually become less distinct from the center to the edges, in the direction perpendicular to the wavefront. For such frontal waves, determining their size is challenging, even for human observers. However, this is not critical when we discuss the variation of wave events based on their locations rather than their sizes.

**Table 1:** Confidence score higher than 0.2

IoU	TP	FP	FN	Precision	Recall	F1 Score	AP
20	575	49	353	92.15%	61.96%	74.10%	83.19%
30	543	81	385	87.02%	58.13%	69.97%	75.38%
40	475	149	453	76.12%	51.19%	61.21%	63.63%
50	398	226	530	63.78%	42.89%	51.29%	48.88%
60	307	317	621	49.20%	33.08%	39.56%	30.74%

#### Example frontal waves images

**Fig. 3:**
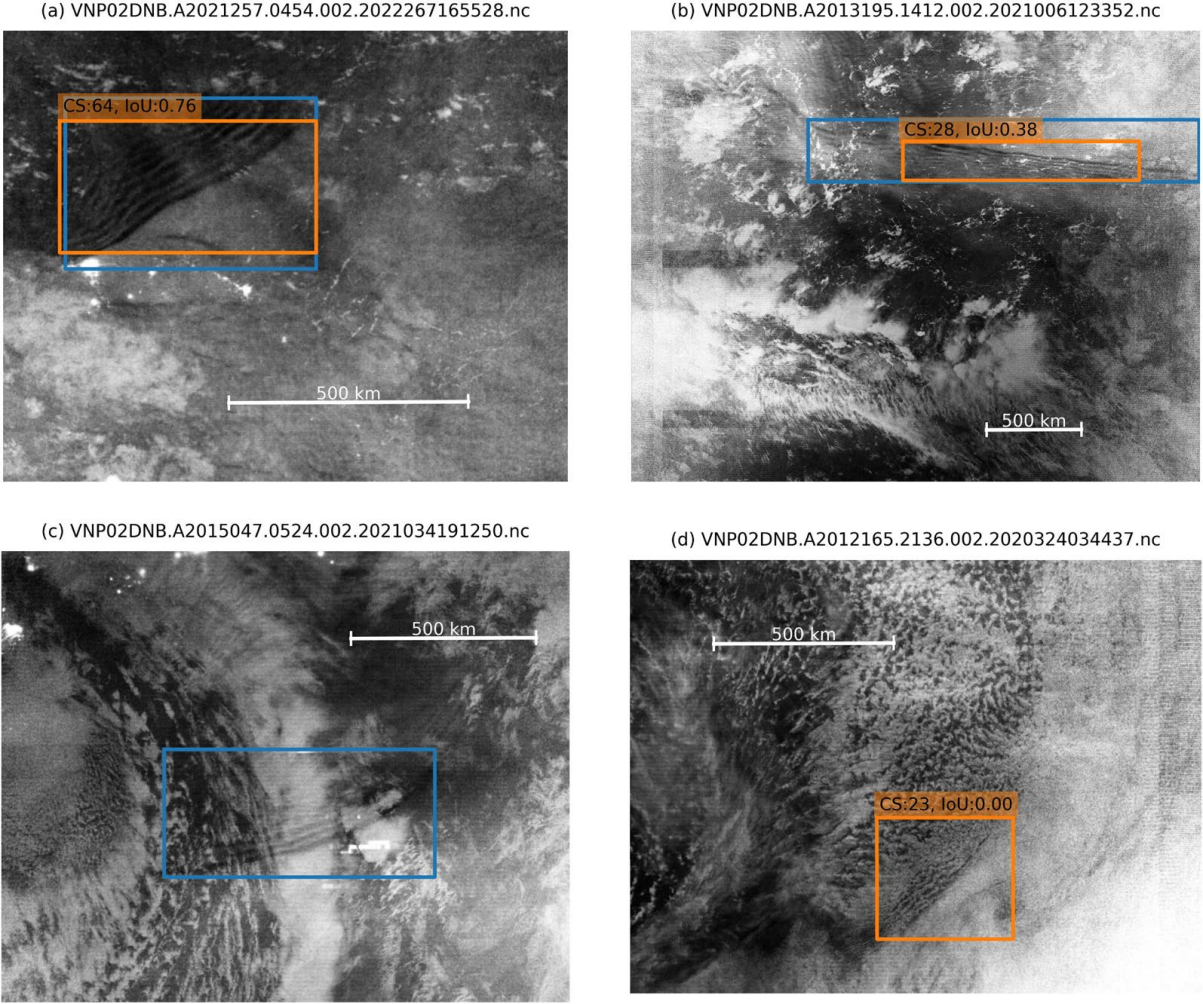
Examples of (**a**) a successfully detected (TP) case, (**b**) a TP, but low IoU case, (**c**) an FN case, and (**d**) an FP case. The name of the original data file is presented on top of each image. Blue rectangles indicate manually labeled bores. Orange rectangles indicate objects detected by the ML model. The confidence score (CS) and IoU are presented at the top of each detected object. Except for (b), all images are cropped to show the object structures larger

Examples of detected frontal waves are presented in Fig. 3. Figure 3(a) shows a successfully detected (TP) case. The manually labeled rectangle (blue) and the ML model-predicted rectangle (orange) overlap well. As a result, the IoU is relatively high. Figure 3(b) also shows a TP case, but the IoU is relatively low. To the human eye, a wavefront aligned in a west-east direction can be recognized from the right edge of the image to about two-thirds of the whole image width. For the ML model, only the center part of the wavefront with relatively sharp undulation can be recognized. Resultantly, the IoU is relatively small at 0.38. Figure 3(c) is a case of FN. A wavefront aligned in a west-east direction is seen by human eyes in the blue rectangle. In this case, tropospheric cloud features overlap over the airglow bore features. A cloud band extended in a north–south direction. Overlapped cloud features contaminate the airglow frontal wave features, and the ML model failed to detect the frontal waves. Figure 3(d) shows a case of FP confused by tropospheric clouds. In the orange rectangle, some features resembling those of frontal waves are seen. A brighter part on the southeast side and a darker part on the northwest are divided by a line at the center. On the darker side, some small-scale undulations are seen. However, the texture of these features is similar to that of tropospheric clouds that one can see outside the orange rectangle. To the eye, they look to be tropospheric cloud patterns. However, the ML model recognizes it is a frontal wave. Not only by clouds, but there are also some FP cases mixed with auroras, ground features such as rivers, or ice on the ocean.

#### Model performance with mass image data

In the test with labeled bore images mentioned in section 4.2, the size of the image set for testing is relatively small and might not accurately reflect the actual distribution of positive and background images. To better assess the model’s performance with a more representative dataset of actual mass data, the trained ML model is applied to all available images of 2012. Note that any data from 2012 was not used in the model training. At the Earth Science Data System, data from day 19 to 366 are published for 2012. Among these, there were 190 moon-free days. To avoid contamination from sunlight, only images with a minimum solar zenith angle of 100 degrees or more were used. There are 15,438 DNB images meeting the criteria. Out of these, 374 objects were classified as positive with a confidence score of 0.2 or higher. Upon manual inspection of all positive images, 199 objects were found to be TP, while the remaining 175 were FP.

**Fig. 4:**
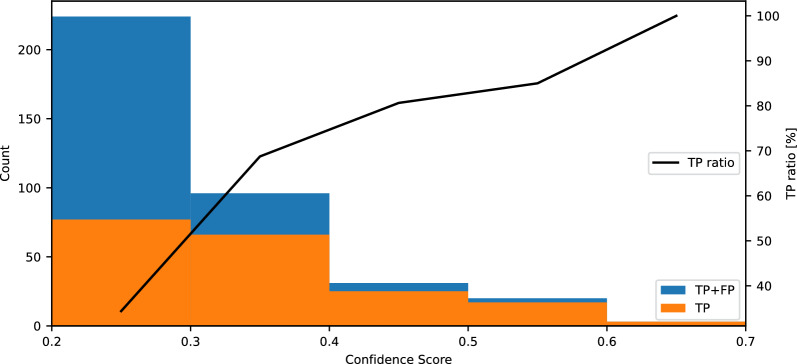
Number of all positive objects (the blue bars) and manually confirmed TP objects (the orange bars) for each confidence score bin. The ratio of TP to all positives, TP/(TP+FP), is presented by the black line

Figure 4 presents histograms of all positive objects and manually confirmed TP objects across various confidence scores. The ratio of TP to all positives (TP/(TP+FP) per bin) is also presented with a black line. This analysis focuses on how different confidence score thresholds affect detection metrics, contrasting with the evaluation of IoU threshold variations with a fixed confidence score threshold discussed in Section 2.1. Notably, the ratio of TP to total positives reaches 100% for confidence scores above 0.6. The ratio of TP gradually decreases as the confidence score decreases. At the bin of confidence score from 0.3 to 0.4, the ratio of TP is about 69%. The 0.2-$-$0.3 bin shows the lowest TP ratio ( 34%), reflecting that the model produces many low-confidence detections, among which FPs are more frequent. But there are still a lot of TPs (77) in the bin.

## Frontal waves variability

To derive eleven years of frontal waves variability, the ML model was applied to the whole available images of Suomi NPP VIIRS/DNB from January 2012 to June 2023. During this period, there were 2,239 moon-free nights and 515,187 images. From these images, the ML model detected 3,283 positive images with a confidence score higher than 0.2. For the variability analysis, only detections with confidence $\ge$ 0.2 were considered. This inclusion threshold is motivated by the performance analysis in the previous section, which shows that (i) TPs are still sufficiently represented at 0.2-0.3, and (ii) the number of detections at $\ge$ 0.2 is manageable for manual verification, whereas lower cutoffs yield many low-yield detections. We conducted a visual survey of the positive images and excluded FP detections and duplicate detections. As a result, 1,150 detected events in 1,121 images were confirmed to be frontal waves. Considering the test result of the model performance that the recall is 61.96%, there should be some missing frontal wave events in the searched images. At the moment, however, with the ML model we trained, this is the largest list of frontal wave events we can obtain. All frontal wave images and the wave event list, which contains the original data file name, geolocation, and observation time, are provided in https://doi.org/10.5281/zenodo.14812061.

**Fig. 5:**
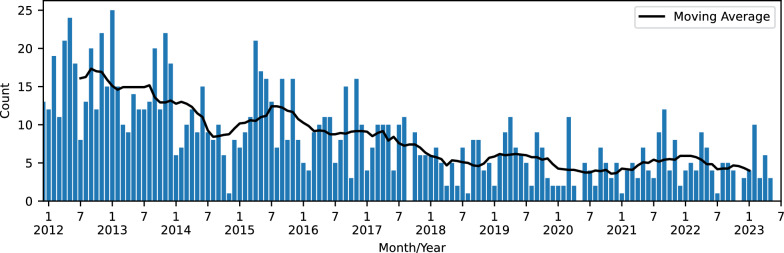
Monthly event counts of detected frontal wave events from January 2012 to June 2023 (the blue bars). The moving average with a window of 12 months is also presented with the black line

Figure 5 shows the time series of monthly numbers of detected frontal wave events from January 2012 to June 2023. The moving average with an average window of 12 months is also presented. The average monthly occurrence gradually decreased over the past eleven years from around 15 in 2012 to around 5 in 2022.

The validity of these long-term trends is difficult to evaluate using only this dataset. As noted above, not only airglow brightness but also background city lights, cloud-top reflections, thin clouds, and instrumental factors affect the preprocessing and the resultant images used for ML-based detection. As shown in Fig. 3(c), factors such as cloud overlap with frontal waves can lead to detection failures. This suggests that other sources of contamination may also contribute to the observed long-term decrease in detected frontal waves. The possibility that instrument factors or data quality influenced the decreasing trend cannot be ruled out, although the sensor’s calibration is monitored and adjusted continuously. This point should be revisited in future studies through detailed analysis using data from longer periods, including multiple solar cycles, and validation against multiple instruments.

Although we cannot rule out the possibility that external factors or instrumental effects have influenced the long-term trends, it is also possible that the observed variations reflect actual changes in wave event occurrence. Over the past 11 years, solar activity has decreased, and CO$_2$ density has increased. These variations influence the middle atmosphere in various parameters, including temperature and wind structures (e.g., Li et al. 2021; Mlynczak et al. 2022; Song and Song 2024, ). The long-term variations of the middle atmosphere may be responsible for the decreasing trend in frontal wave occurrence, although we have no clear explanation of its mechanism at this time. For instance, the Sounding of the Atmosphere using Broadband Emission Radiometry (SABER) observations showed that the upper mesosphere and lower thermosphere have cooled and contracted during the years from 2002 to 2019 (Mlynczak et al. 2022). Song and Song (2024) presented that zonal winds in the mesosphere show long-term trends during the years from 2007 to 2021. Modulations in the thermal structure and wind field can modify the propagation path of gravity waves.

The intensity and peak height of mesospheric airglow could also exhibit long-term variations. Modeling studies have shown that the OH airglow intensity and its peak height are positively correlated with solar activity and negatively correlated with CO$_2$ density (Huang 2016, 2018). This result suggests that the OH airglow has dimmed over the past 11 years, potentially decreasing the visibility of wave signatures in the airglow. Changes in the vertical structure of the airglow can modify its relative height to ducts and affect the visibility of mesospheric bores in the airglow. Further qualitative evaluation is required to assess the impact of these airglow variabilities on the observed variation in mesospheric bore occurrence.

**Fig. 6:**
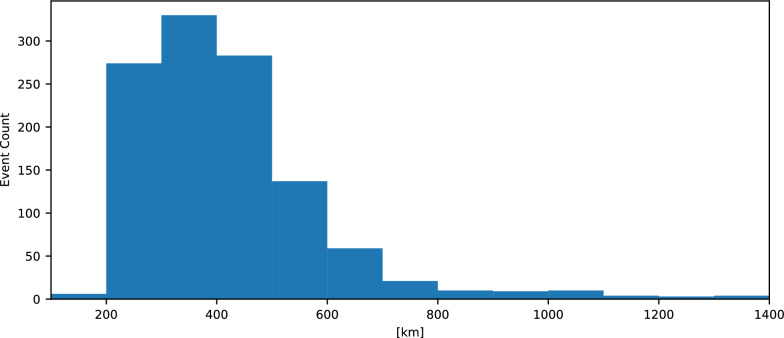
Spatial scale of detected frontal waves. Histogram of the longer side of the detected rectangles

Figure 6 shows the histogram of the longer side of the detected rectangles. Although the ML model’s detection of the edges of frontal waves is not entirely accurate, as discussed in Section 4.1, the histogram provides a rough estimate of the distribution of the spatial scale of frontal wave events. Typically, these events have spatial scales ranging from 200 km to 600 km.

**Fig. 7:**
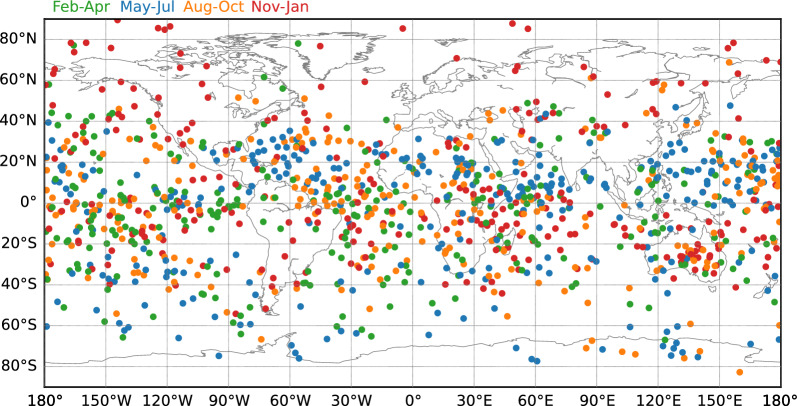
Global distribution of frontal waves from January 2012 to June 2023. Green, blue, orange, and red circles show the geolocations of bores in the months from February to April, May to July, August to October, and November to January, respectively

In Fig. 7, the global distribution of frontal waves is shown. Occurrence in the low latitude is relatively higher. Since the previous global study targeting mesospheric bores from ISS-IMAP/VISI is limited to latitudes within $\pm 55^\circ$ (Hozumi et al. 2019), this is the first comprehensive global view of frontal wave distribution, including high latitudes. There are some frontal wave events at high latitudes over ±60$^\circ$. At mid to low latitudes, especially in the Northern Hemisphere, the occurrence is lower over highly populated areas such as North America, Europe, the Middle East, India, the Indochina peninsula, and China. This is thought to be due to the city light noises that obstruct the airglow observations by DNB. The ML model detector may have missed some airglow frontal wave patterns that overlap with city lights.

**Fig. 8:**
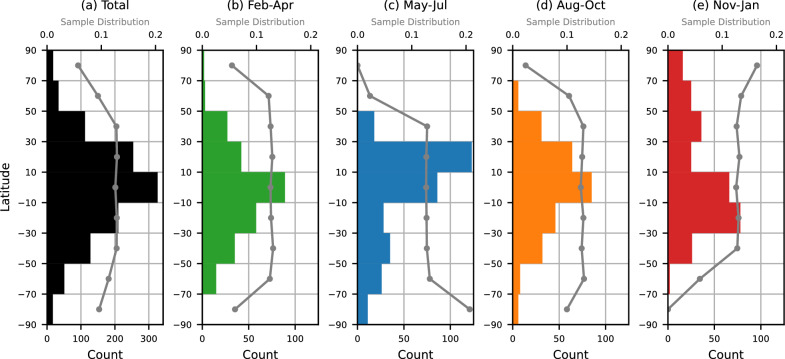
Latitudinal distribution of frontal waves for (**a**) all months from January 2012 to June 2023, (**b**) February to April, (**c**) May to July,** (d**) August to October, and (**e**) November to January. The gray line indicates the empirical sampling distribution across latitude for each season (bin counts normalized to sum to 1)

Figure 8 illustrates the latitudinal distributions of frontal waves. The gray line shows the empirical sampling distribution, defined as the proportion in each latitude bin relative to the seasonal total. The scarcity of samples at winter high latitudes is attributed to shorter nighttime durations. Low latitudes are identified as the most favored regions for bore occurrences across all seasons. In the solstice seasons, there are secondary latitudinal peaks at winter mid-latitudes (30$^\circ$–50$^\circ$N/S).

As mentioned in Section 1, a frontal wave is thought to be a manifestation of a mesospheric bore or a “wall” wave (a large amplitude gravity wave). Low latitudes and winter mid-latitudes are the regions where the migrating diurnal tide (westward propagating diurnal tide with zonal wavenumber 1, hereafter DW1) and the migrating semidiurnal tide (westward propagating semidiurnal tide with zonal wavenumber 2, hereafter SW2) have the largest amplitude in temperature, respectively. In these regions, more or stronger tidally induced mesospheric inversion layers are expected there, and they are thought to make a preferable background environment for bore formation and propagation (Hozumi et al. 2019). The frontal waves detected in this study show higher occurrence in these regions.

Quasi-monochromatic ducted waves could be detected as frontal waves by the ML model. Certain parts of quasi-monochromatic waves observed by imagers are considered to be ducted (Walterscheid et al. 1999; Hecht et al. 2001). The leading edge of such waves may have a similar appearance in airglow to frontal waves. While mesospheric bores have a sharp, step-like jump in airglow brightness, the leading edge of ducted waves should have a more sinusoidal phase front. The preprocessing of the DNB data, including the transformation from an approximate normal distribution to a uniform distribution, does not preserve the linearity of the intensity data and loses fine phase change information. Since the pattern matching of the ML model is based on this preprocessed image, it is difficult to distinguish the minute differences between a step-like jump and a sinusoidal phase front.

Here, we compare our results with previous global studies targeting frontal waves or mesospheric bores (Su et al. 2018; Hozumi et al. 2019). Low-latitude high occurrence of our result is well consistent with the previous studies from DNB Su et al. (2018) and IMAP/VISI. Based on an analysis of the O$_2$(0–0) airglow images of ISS-IMAP/VISI, Hozumi et al. (2019) reported obvious winter mid-latitude peaks of mesospheric bore occurrence that are larger peaks than those at low latitudes in the solstice seasons. The mid-latitude peaks from Suomi NPP VIIRS/DNB are not as prominent and are weaker than those at low latitudes as shown in Fig. 8. This difference would come from the difference in the observing airglows by the two instruments. The DNB observes primarily the OH airglow, whose typical emission peak altitude is 87 km (Sheese et al. 2014). On the other hand, Hozumi et al. (2019) analyzed the O$_2$(0–0) airglow, whose typical emission peak altitude is 95 km (Burrage et al. 1994; Yee et al. 1997). In general, the SW2 peaks at a higher altitude than DW1.

**Fig. 9:**
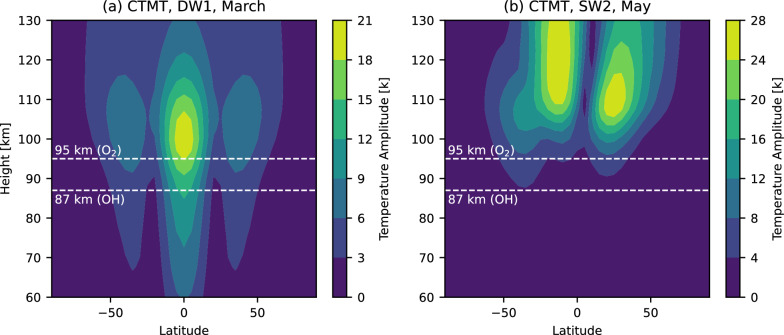
Altitude-latitude profiles of temperature amplitude of the CTMT for (**a**) DW1, March, and (**b**) SW2, May. We selected the months in which DW1 and SW2 each have large amplitudes. Typical emission peak altitudes for the O$_2$(0–0) airglow (95 km) and the OH airglow (87 km) are indicated with the white dashed lines

It is interesting to see how the background conditions differ between the two airglow altitudes. Figure 9 shows altitude-latitude profiles of temperature amplitude of the Climatological Tidal Model of the Thermosphere (CTMT), which is an empirical model of tides (Oberheide et al. 2011). At the altitude of 87 km, DW1 has a substantial amplitude, but SW2 is weaker. In contrast, at 95 km, both DW1 and SW2 display large amplitudes. Consequently, the latitudinal peaks of frontal waves tend to coincide with regions where tidal amplitudes are high, and differences in these background tidal conditions may account for the different latitudinal characteristics of frontal waves observed at the two altitudes. Although local-time sampling is not identical (VISI builds local-time coverage via precession, whereas Suomi NPP samples near 01:30 LT), the stronger winter mid-latitude peaks in VISI can be interpreted primarily as an emission-altitude effect (O$_2$(0–0) $\sim$95 km vs. DNB/OH $\sim$87 km), consistent with the CTMT tidal structure.

## Summary

We trained YOLOv3 to develop a frontal wave detector for the Suomi NPP VIIRS/DNB image. The data set used for the training included manually labeled 696 unique frontal wave images. The trained model was tested on manually labeled 880 images; with an IOU threshold of 0.2, the AP was 83.19%; with an IoU threshold of 0.6, the AP decreased to 30.74%. Although the model is struggling to predict the size of bores, it is able to detect frontal wave events with sufficient accuracy. The model was also tested with 15,438 DNB images, all available images from 2012. The test result showed that above the confidence score of 0.6, 100% of positives are TP.

The model was applied to all the available images of Suomi NPP VIIRS/DNB from January 2012 to June 2023 and detected 3,283 positive images, with an objectivity score higher than 0.2. With a visual survey by human eyes, 1,150 detected events were confirmed to be frontal wave event (TP) out of the positive images. The developed ML model is not a 100% automatic detector, but it is a great helper to conduct a large data set analysis.

With the detected 1,150 frontal wave events, the 11-year variability of frontal waves was studied. The monthly occurrence gradually decreased over the past eleven years from around 15 in 2012 to around 5 in 2022. While we cannot rule out the possibility that external factors or instrumental effects have influenced the long-term trend, this trend needs to be validated against multiple instruments or examined using longer-term data, including multiple solar cycles. Frontal waves have a high occurrence peak at equatorial latitudes and weak occurrence peaks at winter mid-latitudes. Since DW1 and SW2 have large amplitudes in these regions, frontal waves show higher occurrences where temperature inversion layers or wave ducts are more likely to be expected.

## Data Availability

The YOLOv3 code is available from https://github.com/AlexeyAB/darknet. Intensity and geolocation data for the DNB are available from the Level-1 and Atmosphere Archive & Distribution System Distributed Active Archive Center https://ladsweb.modaps.eosdis.nasa.gov/missions-and-measurements/products/VNP02DNB, https://ladsweb.modaps.eosdis.nasa.gov/missions-and-measurements/products/VNP03DNB. Intensity and geolocation data for the M15 band are available from the Level-1 and Atmosphere Archive & Distribution System Distributed Active Archive Center https://ladsweb.modaps.eosdis.nasa.gov/missions-and-measurements/products/VNP02MOD, https://ladsweb.modaps.eosdis.nasa.gov/missions-and-measurements/products/VNP03MOD. Training data, including image files and text files indicating labels, the trained YOLOv3 weights file, the event list and all frontal wave images identified using the ML model are also provided in https://doi.org/10.5281/zenodo.14812061.

## Abbreviations

AP: Average precision
CDF: Cumulative distribution function
CTMT: Climatological tidal model of the thermosphere
DNB: Day/night band
DW1: Migrating diurnal tide (westward-propagating, zonal wavenumber 1)
FN: False negative
FP: False positive
GPU: Graphics processing unit
IMAP: Ionosphere, mesosphere, upper atmosphere and plasmasphere (mission)
IoU: Intersection over union
ISS: International Space Station
L1B: Level-1B (radiometrically calibrated) data
ML: Machine learning
M15: VIIRS mid-infrared band 15
R-CNN: Region-based convolutional neural network
SABER: Sounding of the atmosphere using broadband emission radiometry
SNPP: Suomi National polar-orbiting partnership (Suomi NPP)
SSD: Single Shot MultiBox detector
SW2: Migrating semidiurnal tide (westward-propagating, zonal wavenumber 2)
TP: True positive
VIIRS: Visible/Infrared imaging radiometer suite
VISI: Visible and near-Infrared Spectral Imager
YOLO: “You Only Look Once” (object-detection framework)
YOLOv3: You Only Look Once, version 3
